# Construction of a multifactorial prediction model for healthcare workers’ work ability: focusing on the interaction and impact pathways of job burnout and sleep disorder

**DOI:** 10.3389/fpubh.2026.1787439

**Published:** 2026-03-17

**Authors:** Ni Wang, Liang Shang, Ting Zhou

**Affiliations:** Xi Yuan Second Ward, Shaanxi Provincial People's Hospital, Xi’an, Shaanxi, China

**Keywords:** additive interaction, healthcare workers, job burnout, latent profile analysis, sleep quality, structural equation modeling, work ability

## Abstract

**Objective:**

To explore the latent profiles, core associated factors, and complex mechanisms of work ability among healthcare workers in large tertiary hospitals in China.

**Methods:**

A cross-sectional study was conducted from July to October 2025. A convenience sample of 1,590 healthcare workers from a large tertiary hospital in Shaanxi Province was assessed using the Work Ability Index (WAI), the Maslach Burnout Inventory-General Survey (MBI-GS), and the Pittsburgh Sleep Quality Index (PSQI). Latent profile analysis (LPA) was employed to identify potential categories of work ability. Multivariable logistic regression analysis was performed to determine independently associated factors and to construct a nomogram prediction model. An additive interaction model and structural equation modeling (SEM) were used to analyze the joint effect and the influential pathways of job burnout and sleep disorder.

**Results:**

LPA identified two distinct categories: “Good Work Ability” (73%) and “Poor Work Ability” (27%). Multivariable regression analysis indicated that job burnout (OR = 3.770, 95% CI: 2.510–5.661) and sleep disorder (OR = 2.890, 95% CI: 2.121–3.939) were the factors most strongly associated with poor work ability. Longer working years (≥21 years) and higher professional titles (intermediate/senior) were also associated with an increased likelihood of poor work ability. In contrast, higher education (master’s degree or above) and regular physical exercise were associated with a decreased likelihood. The predictive nomogram model demonstrated good discriminative ability (AUCs of 0.781 and 0.740 for the training and validation sets, respectively) and clinical utility. Interaction analysis revealed a significant positive additive interaction between job burnout and sleep disorder (RERI = 5.164, AP = 47.453%). SEM supported a model in which job burnout was not only directly and negatively associated with work ability (*β* = −0.359, *p* < 0.01) but also showed an indirect association via impaired sleep quality (*β* = −0.281, *p* < 0.01).

**Conclusion:**

Among healthcare workers in large tertiary hospitals in China, job burnout and sleep disorder are two core and synergistic factors associated with work ability. The prediction model based on multiple factors can provide a practical tool for the early identification of high-risk individuals. Future occupational health intervention programs need to adopt integrated strategies, targeting both the alleviation of job burnout and the improvement of sleep quality as dual core objectives, and implement precise prevention and control for key populations such as those with long service years and high professional titles to maintain and enhance the work ability of healthcare workers.

## Background

The work ability of healthcare workers constitutes the fundamental cornerstone for ensuring the efficiency of the healthcare system and patient safety. Globally, the healthcare industry is commonly confronted with issues such as escalating workload, strained human resources, and resultant occupational health problems (1, 2)^.^ In China, the vast healthcare service system bears tremendous demand, with healthcare workers, particularly those in large tertiary A-level hospitals, enduring long-term high-pressure work environments (3). This reality poses a severe challenge not only to their physical and mental health but is also directly linked to the operational efficiency of the healthcare system and patient safety (4). Against this backdrop, maintaining and enhancing the work ability of healthcare workers has become a critical issue in the field of occupational health.

Work ability refers to an individual’s comprehensive state of meeting specific job requirements in terms of physical, psychological, and professional skills. It is not a static trait but rather a result of dynamic equilibrium, profoundly dependent on the ongoing interaction between personal resources and job demands as well as occupational pressures (5). The Conservation of Resources (COR) theory provides a robust framework for understanding this dynamic process. This theory posits that individuals strive to obtain, retain, protect, and foster their valuable resources (e.g., energy, positive affect, sleep health); psychological stress and negative consequences arise when these resources are threatened with loss, are actually lost, or when investment in resources fails to yield adequate returns (6, 7). From this perspective, job burnout is essentially a state of chronic depletion of key psychological resources such as emotional and cognitive reserves, while sleep disorder signifies an impairment of the fundamental resource-replenishing processes of physiological restoration and cognitive consolidation. Both can be viewed as severe depletion of an individual’s core resources and may trigger a “loss spiral”—where initial resource loss weakens the individual’s capacity to cope with subsequent demands, leading to further resource depletion, thereby exerting a cumulative negative impact on work ability (7).

Among the numerous factors affecting the work ability of healthcare workers, job burnout has been widely substantiated as a core psychosocial risk factor (8, 9). Since its introduction by Freudenberger, burnout has been clearly defined as a psychological syndrome resulting from chronic workplace stress, characterized by emotional exhaustion, depersonalization, and reduced personal accomplishment (10, 11). Due to its inherent characteristics of high responsibility, substantial emotional investment, and significant uncertainty, the healthcare industry has become a high-prevalence field for burnout. A systematic review reports that the prevalence of burnout among healthcare workers in various settings ranges from 30 to 50% (12). Burnout is not merely a distressing experience at the individual level; it directly erodes work ability. By persistently depleting cognitive resources, undermining intrinsic work motivation, and impairing the accuracy of clinical judgment, it ultimately leads to decreased work efficiency and increased error rates, constituting a key psychological pathway to the impairment of work ability.

In addition to job burnout, sleep disorder represents another prevalent issue, and the two are often interrelated. Shift work, irregular schedules, and chronic psychological stress are primary factors disrupting the sleep–wake rhythm and quality among healthcare workers (13). The pervasiveness of this problem is well-documented; for instance, a Chinese cross-sectional study reported a 52.7% prevalence of sleep disorders among clinical nurses (14). As a vital physiological restorative process, sleep quality is directly linked to daytime cognitive function, emotional state, and physical energy levels. For clinical work that demands high concentration and rapid response, sleep disorders impair executive function, memory, and operational precision, thereby undermining work ability at a physiological level. This impairment may not only lead to the accumulation of fatigue but could also exacerbate job burnout, forming a mutually reinforcing negative cycle (15).

Although the high prevalence of job burnout and sleep disorders, along with their respective negative correlations with work ability, are widely acknowledged, the intrinsic mechanisms of how they jointly influence this core occupational metric remain unclear, and current research exhibits limitations. Firstly, most studies treat work ability as a homogeneous entity or a simple continuum, overlooking its potential heterogeneous structure, which hinders the precise identification of high-risk subgroups. Secondly, existing research predominantly analyses job burnout and sleep disorders as independent factors, failing to adequately reveal their complex joint pattern of impact. Specifically, two key questions warrant exploration: First, is their impact on work ability a simple additive risk or does a synergistic (‘1 + 1 > 2’) interaction effect exist? Based on the Conservation of Resources theory, when job burnout (depletion of psychological resources) and sleep disorder (impairment of physiological restorative resources) coexist, they may inflict a disproportionate, compounded blow to work ability due to a “loss spiral” (7). Some empirical studies provide corroborative evidence, suggesting that sleep quality may moderate the relationship between stressors and occupational fatigue, and that sleep problems can exacerbate cognitive failures in the workplace (16, 17). Second, can the impact of job burnout be realized through the mediating pathway of ‘affecting sleep quality’? This aligns with the notion of “resource loss spirals,” where the loss of one core resource (psychological resources) leads to the loss of another linked resource (sleep restorative capacity) (18).

Finally, at the practical level, there is a lack of clinical tools capable of integrating these core factors for individualized risk assessment. To address these gaps, this study aims to: (1) utilize Latent Profile Analysis (LPA) to uncover the heterogeneous structure of work ability; (2) construct and validate a multifactorial clinical prediction model (nomogram); and (3) employing the Conservation of Resources theory as a framework, use an additive interaction model and structural equation modeling (SEM) to, respectively, examine the synergistic interaction effect of job burnout and sleep disorder on work ability, as well as the mediating pathway of ‘job burnout → sleep quality → work ability.’ This is intended to provide an integrated theoretical explanation and practical tools for precision occupational health management.

To address these deficiencies, this study employs LPA. Based on individuals’ response patterns across the seven dimensions of the Work Ability Index (WAI), LPA objectively identifies latent subgroups with similar characteristics, thereby revealing the heterogeneous structure of work ability. All seven WAI dimensions were selected as indicators because they conceptually represent distinct yet interrelated facets of overall work ability (e.g., current work ability compared with lifetime best, work ability in relation to job demands, number of current diseases diagnosed by a physician, estimated work impairment due to diseases, sick leave during the past year, prognosis of work ability two years from now, and psychological resources) (19). These dimensions collectively assess an individual’s resources pertinent to their work, aiding in capturing the multidimensional heterogeneity of work ability and identifying subgroups with distinct profiles of personal resource configurations (e.g., an employee may possess strong psychological resources and a positive future prognosis but simultaneously suffer from poor physical health). Compared to the arbitrary dichotomization based on a total score, LPA determines the optimal classification through statistical modeling, supplemented by indices such as entropy to evaluate classification quality. This approach ensures that the identification results are empirically driven, replicable, and exhibit good between-group discrimination, thereby establishing a reliable methodological foundation for subsequent analyses.

Based on this, the specific objectives of this study are as follows:

(1) To identify latent heterogeneous categories of healthcare workers’ work ability using LPA;
(2) To construct and validate a multifactorial clinical prediction model based on the identified categories and core risk factors, and to translate it into a user-friendly graphical risk assessment tool for clinical application;
(3) To explore the dual statistical relationships of job burnout and sleep disorder with work ability: examining synergistic effects using an additive interaction model and analyzing the fit of the hypothesized pathway ‘job burnout → sleep quality → work ability’ through SEM.

The findings of this study are expected to provide crucial correlational evidence and a readily applicable assessment tool for establishing a precision occupational health management system with dual targets of alleviating burnout and improving sleep.

## Materials and methods

### Study design and data collection

This cross-sectional study strictly adhered to the Strengthening the Reporting of Observational Studies in Epidemiology (STROBE) guidelines (20). A convenience sampling method was employed to select currently employed healthcare workers from a large tertiary hospital in Shaanxi Province, China.

The sample size was estimated based on the formula for estimating a population mean in a cross-sectional study: $\left[n={\left(\frac{z_{1-\alpha /2}\times \sigma }{\delta }\right)}^{2}\right]$ . The significance level (*α*) was set at 0.05 (two-tailed), with *Z*₁–*α*/₂ = 1.96. The parameter *σ*, representing the standard deviation of the primary outcome measure (the Work Ability Index score), was set at 4.8 based on a previous study utilizing the same scale among hospital staff (21). The margin of error (*δ*) was set at 1.0 point. The calculated minimum theoretical sample size was approximately 89 participants. To account for the design effect associated with convenience sampling and potential invalid questionnaires, the sample size was increased by 50%, resulting in an initially planned sample size of 134 participants. However, given that this study planned to employ multiple complex statistical methods, including LPA, multivariable logistic regression, and SEM, the sample size required in practice should be significantly larger than that for conventional cross-sectional surveys to ensure the stability of model fit, statistical power, and the reliability of the results. Referring to methodological literature, for conducting multivariate analyses involving latent variables (such as LPA and SEM), a sample size of 500–1,000 is typically recommended (22, 23)^.^ Particularly for the stable testing of interaction and mediation effects, a larger sample size contributes to improving the precision of parameter estimates and statistical power (24). Therefore, this study set a higher target for sample collection in the actual survey (>1,500 cases), aiming to provide a sufficient statistical foundation for all analytical steps. Ultimately, a total of 1700 questionnaires were distributed, with 1,655 returned, yielding a response rate of 97.35%. Following rigorous quality control checks, 65 invalid questionnaires exhibiting patterned responses, obvious logical inconsistencies, or severe missing key information were excluded. Consequently, 1,590 valid questionnaires were included for analysis, resulting in an effective response rate of 96.07%.

All participants were fully informed of the study’s purpose, procedures, and their rights prior to the survey and provided written informed consent. Questionnaires were completed anonymously. For questionnaires with 1–2 missing items, the mean score of that specific item from the sample was used for imputation. Questionnaires with more than 3 missing items were excluded. The specific inclusion criteria were as follows: (1) holding valid professional licensure and registration as a physician, nurse, or medical technician and being currently employed in the role; (2) having worked in a frontline clinical position for ≥1 year; (3) possessing the ability to independently read, comprehend, and complete an electronic questionnaire; (4) not currently taking medication for any psychiatric or psychological disorder; and (5) voluntary participation in this study. Exclusion criteria were: (1) not being a formal hospital employee (e.g., interns, visiting trainees); (2) being on long-term leave, out for training, or otherwise absent from duty during the survey period; and (3) refusal to participate or failure to complete the questionnaire.

## Methods

### Basic information

The survey collected data on gender, age, occupational category, education level, years of work experience, professional title, marital status, monthly income, shift schedule, presence of chronic diseases, and exercise (per week).

### Job burnout

Job burnout was assessed using the Maslach Burnout Inventory-General Survey (MBI-GS) (25, 26). This study utilized the Chinese version of the MBI-GS, which was translated and revised by Li Chaoping. This version has undergone cross-cultural adaptation for Chinese healthcare practitioners and demonstrates good applicability (27). This scale consists of 15 items covering three dimensions: emotional exhaustion (5 items), cynicism (4 items), and reduced professional efficacy (6 items). Each item is measured using a 7-point Likert scale (0 = “never” to 6 = “always”). A composite score was calculated using the formula: Total score = 0.4 × mean Emotional Exhaustion score + 0.3 × mean Cynicism score + 0.3 × mean Professional Efficacy score. A total score of 0–1.49 indicates no burnout, 1.50–3.49 indicates mild to moderate burnout, and 3.50–6.00 indicates severe burnout (28, 29). In this study, the Cronbach’s *α* coefficient for this scale was 0.833.

### Sleep quality

Subjective sleep quality over the past month was assessed using the Pittsburgh Sleep Quality Index (PSQI) (30). This study employed the Chinese version of PSQI, which was revised by Liu Xianchen et al. (31). This scale comprises seven components: subjective sleep quality, sleep latency, sleep duration, sleep efficiency, sleep disturbances, use of sleep medication, and daytime dysfunction. The global score ranges from 0 to 21, with a higher score indicating poorer sleep quality. Based on the domestic standard (32), a global score >7 was used to define the presence of a sleep disorder. In this study, the Cronbach’s *α* coefficient for this scale was 0.872.

### Work ability evaluation

The Work Ability Index (WAI) questionnaire was developed over several years by the Finnish Institute of Occupational Health in 1994 (33). This study employed the Chinese version of the WAI, which was revised by Zhang Lei et al. (34). This scale includes 7 items (comprising a total of 10 questions) covering physical, mental, and illness-related aspects. The scale score is the sum of the 7 item scores, ranging from 7 to 49, with a higher score indicating better work ability. In this study, the Cronbach’s *α* coefficient for this scale was 0.703.

### Quality control

To ensure data quality, multiple measures were implemented during both data collection and post-collection stages. First, during the online survey, technical settings on the “Wenjuanxing” platform were applied: (1) each questionnaire link could only be accessed once per IP address to prevent duplicate submissions from the same network; (2) mandatory answering was set for all items to avoid missing data. Second, after data collection, a dedicated team of three investigators (all with training in medical research methods) performed a two-step manual review. Invalid questionnaires were identified and excluded based on the following pre-defined criteria: (1) patterned responses (e.g., straight-lining, alternating extreme values like “1, 6, 1, 6…”); (2) obvious logical inconsistencies (e.g., reporting “no diagnosed disease” but then listing multiple sick leave days); (3) implausibly short completion time (less than one-third of the estimated average time). All participants were fully informed about the study’s purpose, procedures, and their rights prior to data collection and provided signed informed consent. To protect privacy, the study employed anonymous questionnaires and did not collect any personally identifiable information (such as name, ID number, etc.).

### Statistical analysis

LPA was conducted using Mplus version 8.3. The optimal number of classes was determined by comparing model fit indices across different class solutions, including the Akaike Information Criterion (AIC), the Bayesian Information Criterion (BIC), the sample size-adjusted Bayesian Information Criterion (aBIC), entropy, and the results of the Bootstrapped Likelihood Ratio Test (BLRT). Lower values of AIC, BIC, and aBIC indicate better model fit. The BLRT was used to compare the fit between a model with k classes and a model with *k* – 1 classes; a significant result (*p* < 0.05) suggests that the k-class model provides a superior fit. Entropy, which ranges from 0 to 1, was used to evaluate the accuracy of classification, with values closer to 1 indicating more precise class separation (35).

Data analysis was performed using R Studio version 4.2.2. Categorical variables were described using frequencies (*N*) and percentages (%), while continuous variables were presented as mean ± standard deviation ( $\bar{x}$ ± s). Comparisons between two group means were conducted using the independent samples *t*-test, and comparisons across multiple groups were performed using one-way analysis of variance (ANOVA). Using the latent classes of work ability as the dependent variable, multivariable logistic regression analysis was employed to identify influencing factors. To develop a clinical prediction tool, a nomogram model was constructed and internally validated. The dataset was randomly split into a training set and a validation set at a 7:3 ratio. The discrimination ability of the model was evaluated by plotting the Receiver Operating Characteristic (ROC) curve and calculating the Area Under the Curve (AUC). Calibration was assessed using calibration curves, and clinical utility was evaluated via Decision Curve Analysis (DCA). The additive interaction model was applied to assess the joint effect of job burnout and sleep disorder on work ability. The Relative Excess Risk due to Interaction (RERI), the Attributable Proportion due to Interaction (AP), and the Synergy Index (S) along with their 95% confidence intervals (95% CI) were calculated. An additive interaction was considered statistically significant if the 95% CI for RERI or AP did not include 0, or if the 95% CI for S did not include 1 (36).

SEM analysis was performed using AMOS 26.0 to examine the pathways among nurses’ job burnout, sleep quality, and work ability. The model fit was assessed using the following indices: the chi-square to degrees of freedom ratio (*χ*^2^/df), the Root Mean Square Error of Approximation (RMSEA), the Goodness-of-Fit Index (GFI), the Adjusted Goodness-of-Fit Index (AGFI), the Normed Fit Index (NFI), the Tucker-Lewis Index (TLI), the Incremental Fit Index (IFI), and the Comparative Fit Index (CFI). The criteria for an acceptable model fit were generally set as: *χ*^2^/df < 3, RMSEA < 0.08, and values for GFI, AGFI, NFI, TLI, IFI, and CFI all greater than 0.90 (37). The Bollen–Stine Bootstrap method was employed to correct the model fit.

## Results

### General characteristics

This study included a total of 1,590 healthcare workers. The participants were predominantly female (74.65%) and aged under 30 years (51.14%). Regarding occupational composition, nurses constituted the largest proportion (50.50%), followed by physicians (35.60%) and medical technicians (13.90%). The vast majority of respondents held a bachelor’s degree or higher (95.09%), held a primary professional title (75.91%), and were required to participate in shift work (69.12%). In terms of health-related aspects, most participants had no history of chronic diseases (93.96%) and engaged in physical exercise 1–2 times per week (46.04%). Among the core variables of interest in this study, the prevalence of job burnout was 68.30%, and the prevalence of sleep disorder was 39.37% (Table 1).

**Table 1:** Sociodemographic characteristics and distribution of core variables of the study participants (*N* = 1,590).

Variables	Category	Total	%
Gender	Male	403	25.35
	Female	1,187	74.65
Age (years)	<30	813	51.14
	30–39	533	33.52
	40–49	170	10.69
	≥50	74	4.65
Occupation category	Physician	566	35.60
	Nurse	803	50.50
	Medical technician	221	13.90
Education	Associate degree or below	78	4.91
	Bachelor’s degree	684	43.01
	Master’s degree	780	49.06
	Doctoral degree or above	48	3.02
Working years	≤5	734	46.16
	6–10	369	23.21
	11–20	276	17.36
	21–30	153	9.62
	≥31	58	3.65
Professional title	Junior	1,207	75.91
	Intermediate	275	17.30
	Senior	108	6.79
Chronic disease	No	1,494	93.96
	Yes	96	6.04
Marital status	Unmarried	619	38.93
	Married	971	61.07
Monthly income (CNY)	<10,000	683	42.96
	≥10,000	907	57.04
Shift work	Fixed day shift	491	30.88
	Rotating shifts	1,099	69.12
Exercise (per week)	None	504	31.70
	1–2 times	732	46.04
	3–4 times	253	15.91
	≥5 times	101	6.35
Job burnout	No	504	31.70
	Yes	1,086	68.30
Sleep disorder	No	964	60.63
	Yes	626	39.37

### LPA of healthcare workers’ WAI

**Figure 1:**
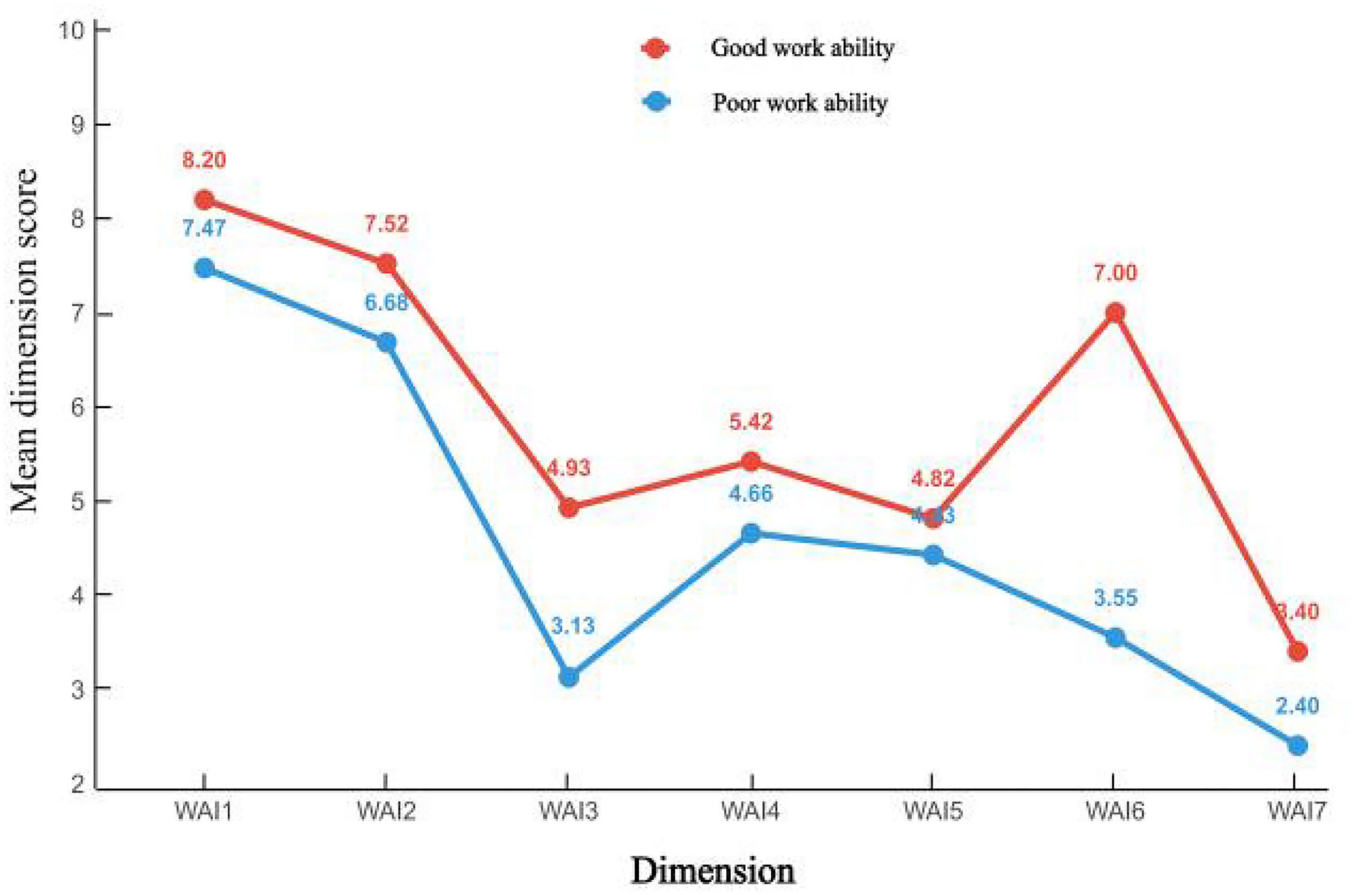
Characteristics of the latent profiles of WAI among healthcare workers. The dimensions of the work ability index (WAI) are defined as follows: WAI1 (current work ability compared with lifetime best); WAI2 (work ability in relation to the demands of the job); WAI3 (number of current diseases diagnosed by a physician); WAI4 (estimated work impairment due to illness); WAI5 (sick leave during the past 12 months); WAI6 (personal prognosis of work ability 2 years from now); WAI7 (mental resources).

Using the seven dimensions of the WAI as observed variables, latent profile models with 1 to 3 classes were sequentially fitted. The fit indices for each model are presented in Table 2. The AIC, BIC, and SABIC values all gradually decreased as the number of latent classes increased. The two-class model demonstrated perfect classification accuracy (Entropy = 1.000), and the BLRT result supported this solution (*p* = 0.01). In contrast, while the three-class model showed lower AIC/BIC values, its classification accuracy was reduced (Entropy = 0.861), and its smallest class comprised only a very small proportion of the sample (approximately 6%). Given the superior classification clarity of the two-class solution and considering the parsimony and stability required for subsequent analyses, the two-class model was determined to be optimal. The estimated latent class probabilities were 0.27 and 0.73, and the two classes were accordingly named “Poor Work Ability” and “Good Work Ability.” As shown in the profile characteristics of the two latent classes (Figure 1), scores on all seven dimensions of the WAI were higher in the “Good Work Ability” group compared to the “Poor Work Ability” group.

**Table 2:** Fit indices for the LPA models with different numbers of classes.

Classes	AIC	BIC	SABIC	Entropy	BLRT value	BLRT *p*-value	Class probabilities
1	38,922.67	38,997.99	38,953.51	1.000	NA	NA	–
2	36,354.59	36,472.94	36,403.05	1.000	2,584.081	0.01	0.27/0.73
3	35,916.71	36,078.1	35,982.79	0.861	453.88	0.01	0.06/0.70/0.23

### Basic characteristics of healthcare workers by work ability Group

Univariate analysis (Table 3) revealed statistically significant differences (all *p* < 0.05) in the distributions of age, education level, working years, professional title, chronic disease status, exercise (per week), job burnout, and sleep disorder between the different work ability groups. Specifically, the poor work ability group had higher proportions of individuals aged ≥40 years, with working years ≥21 years, holding intermediate or senior professional titles, having chronic diseases, lacking exercise habits, and experiencing job burnout and sleep disorder. In contrast, the good work ability group had higher proportions of individuals with a master’s degree or higher and those exercising ≥3 times per week. The distributions of gender, occupational category, monthly income, and shift work system showed no statistically significant differences between the two groups (all *p* > 0.05).

**Table 3:** Comparison of basic characteristics between healthcare worker groups with different work ability levels [*n* (%)].

Variables	Total (*n* = 1,590)	Good work ability (*n* = 1,164)	Poor work ability (*n* = 426)	*χ* ^2^	*p*
Gender, *n* (%)				2.43	0.119
Male	403 (25.35)	307 (26.37)	96 (22.54)		
Female	1,187 (74.65)	857 (73.63)	330 (77.46)		
Age, *n* (%)				57.24	<0.001
<30 years	813 (51.13)	623 (53.52)	190 (44.60)		
30–39 years	533 (33.52)	403 (34.62)	130 (30.52)		
40–49 years	170 (10.69)	109 (9.36)	61 (14.32)		
≥50 years	74 (4.65)	29 (2.49)	45 (10.56)		
Occupation category, *n* (%)				3.32	0.190
Physician	566 (35.60)	427 (36.68)	139 (32.63)		
Nurse	803 (50.50)	584 (50.17)	219 (51.41)		
Medical technician	221 (13.90)	153 (13.14)	68 (15.96)		
Education, *n* (%)				22.57	<0.001
Associate degree or below	78 (4.91)	40 (3.44)	38 (8.92)		
Bachelor’s degree	684 (43.02)	501 (43.04)	183 (42.96)		
Master’s degree	780 (49.06)	583 (50.09)	197 (46.24)		
Doctoral degree or above	48 (3.02)	40 (3.44)	8 (1.88)		
Working years, *n* (%)				70.90	<0.001
≤5	734 (46.16)	570 (48.97)	164 (38.50)		
6–10	369 (23.21)	291 (25.00)	78 (18.31)		
11–20	276 (17.36)	196 (16.84)	80 (18.78)		
21–30	153 (9.62)	82 (7.04)	71 (16.67)		
≥31	58 (3.65)	25 (2.15)	33 (7.75)		
Professional title, *n* (%)				47.47	<0.001
Junior	1,207 (75.91)	927 (79.64)	280 (65.73)		
Intermediate	275 (17.30)	185 (15.89)	90 (21.13)		
Senior	108 (6.79)	52 (4.47)	56 (13.15)		
Chronic disease, *n* (%)				33.32	<0.001
No	1,494 (93.96)	1,118 (96.05)	376 (88.26)		
Yes	96 (6.04)	46 (3.95)	50 (11.74)		
Marital status, *n* (%)				3.83	0.050
Unmarried	619 (38.93)	470 (40.38)	149 (34.98)		
Married	971 (61.07)	694 (59.62)	277 (65.02)		
Monthly income (CNY), *n* (%)				0.12	0.732
<10,000	683 (42.96)	503 (43.21)	180 (42.25)		
≥10,000	907 (57.04)	661 (56.79)	246 (57.75)		
Shift work, *n* (%)				0.64	0.422
Fixed day shift	491 (30.88)	366 (31.44)	125 (29.34)		
Rotating shifts	1,099 (69.12)	798 (68.56)	301 (70.66)		
Exercise (per week), *n* (%)				46.78	<0.001
None	504 (31.70)	314 (26.98)	190 (44.60)		
1–2 times	732 (46.04)	567 (48.71)	165 (38.73)		
3–4 times	253 (15.91)	198 (17.01)	55 (12.91)		
≥5 times	101 (6.35)	85 (7.30)	16 (3.76)		
Job burnout, *n* (%)				112.19	<0.001
No	504 (31.70)	456 (39.18)	48 (11.27)		
Yes	1,086 (68.30)	708 (60.82)	378 (88.73)		
Sleep disorder, *n* (%)				137.79	<0.001
No	964 (60.63)	807 (69.33)	157 (36.85)		
Yes	626 (39.37)	357 (30.67)	269 (63.15)		

### Multivariable logistic regression analysis of factors associated with healthcare workers’ work ability

Multivariable logistic regression analysis, with work ability status (1 = Poor, 0 = Good) as the dependent variable, showed (Table 4) that job burnout (OR = 3.770, 95% CI: 2.510–5.661) and sleep disorder (OR = 2.890, 95% CI: 2.121–3.939) were positively associated with poor work ability. Regarding working years, compared to those with ≤5 years, working 6–10 years was negatively associated with poor work ability (OR = 0.647, 95% CI: 0.425–0.986), while working 21–30 years (OR = 2.417, 95%CI: 1.345–4.344) and ≥31 years (OR = 2.548, 95% CI: 1.116–5.818) were positively associated. Working 11–20 years showed no significant association (*p* > 0.05). Furthermore, compared to junior professional titles, intermediate (OR = 1.789, 95% CI: 1.132–2.829) and senior titles (OR = 2.134, 95% CI: 1.091–4.175) were positively associated with poor work ability. Regarding exercise frequency, compared to no exercise, exercising 1–2 times per week (OR = 0.570, 95% CI: 0.407–0.798) and ≥5 times per week (OR = 0.287, 95% CI: 0.128–0.642) were negatively associated with poor work ability. In terms of education level, compared to those with a college degree or below, a master’s degree (OR = 0.371, 95% CI: 0.195–0.708) and a doctoral degree or above (OR = 0.216, 95% CI: 0.068–0.684) were negatively associated with poor work ability.

**Table 4:** Multivariable logistic regression analysis of factors associated with healthcare workers’ work ability.

Variables	*β*	SE	*Z*	*p*	OR (95% CI)
Intercept	−1.537	0.385	−3.993	<0.001	0.215 (0.101–0.457)
Education					
Associate degree or below					1.000 (Reference)
Bachelor’s degree	−0.631	0.324	−1.95	0.051	0.532 (0.282–1.003)
Master’s degree	−0.99	0.329	−3.011	0.003	0.371 (0.195–0.708)
Doctoral degree or above	−1.531	0.587	−2.607	0.009	0.216 (0.068–0.684)
Working years					
≤5					1.000 (Reference)
6–10	−0.435	0.215	−2.026	0.043	0.647 (0.425–0.986)
11–20	−0.057	0.245	−0.234	0.815	0.944 (0.585–1.525)
21–30	0.883	0.299	2.951	0.003	2.417 (1.345–4.344)
≥31	0.935	0.421	2.22	0.026	2.548 (1.116–5.818)
Professional title					
Junior					1.000 (Reference)
Intermediate	0.582	0.234	2.489	0.013	1.789 (1.132–2.829)
Senior	0.758	0.342	2.214	0.027	2.134 (1.091–4.175)
Exercise (per week)					
None					1.000 (Reference)
1–2 times	−0.563	0.172	−3.269	0.001	0.570 (0.407–0.798)
3–4 times	−0.411	0.238	−1.725	0.085	0.663 (0.416–1.058)
≥5 times	−1.249	0.411	−3.039	0.002	0.287 (0.128–0.642)
Job burnout					
No					1.000 (Reference)
Yes	1.327	0.207	6.396	<0.001	3.770 (2.510–5.661)
Sleep disorder					
No					1.000 (Reference)
Yes	1.061	0.158	6.721	<0.001	2.890 (2.121–3.939)

### Construction of the nomogram prediction model

**Figure 2:**
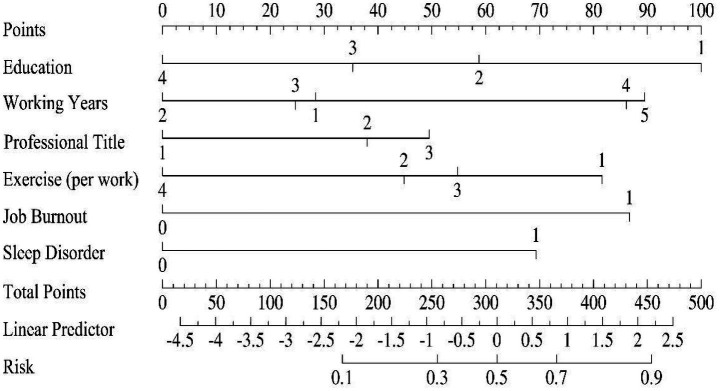
Nomogram for predicting the risk of poor work ability among healthcare workers. Nomogram model for predicting the risk of declined work ability among healthcare workers. The numbers below each variable in the figure represent the coding for that variable: Education level: 1 = college or below, 2 = bachelor’s degree, 3 = master’s degree, 4 = doctoral degree or above; Working years: 1 = ≤5, 2 = 6–10, 3 = 11–20, 4 = 21–30, 5 = ≥31; Professional title: 1 = junior, 2 = intermediate, 3 = senior; exercise (per week): 1 = none, 2 = 1–2 times, 3 = 3–4 times, 4 = ≥5 times; Job burnout: 0 = no, 1 = yes; Sleep disorder: 0 = no, 1 = yes. To use it, locate the individual’s value on each corresponding variable axis, draw a line vertically upward to align with the “Points” axis to read the score for each variable, sum them to obtain the total points, then project this total vertically downward to the bottom “Risk” axis to obtain the predicted probability of declined work ability for that individual.

Based on the results of the multivariable logistic regression analysis, a nomogram model was constructed to predict the risk of poor work ability. This model incorporates six predictive factors: education level, working years, professional title, exercise (per week), job burnout, and sleep disorder. Different values for each variable correspond to specific point scores (Points). To use the model, the point scores corresponding to an individual’s characteristics for each variable are obtained and summed to yield a total point score (Total Points). Projecting this total score downward to the bottom Linear Predictor axis and the Risk axis provides an individualized predicted probability of poor work ability (Figure 2).

### Model validation

**Figure 3:**
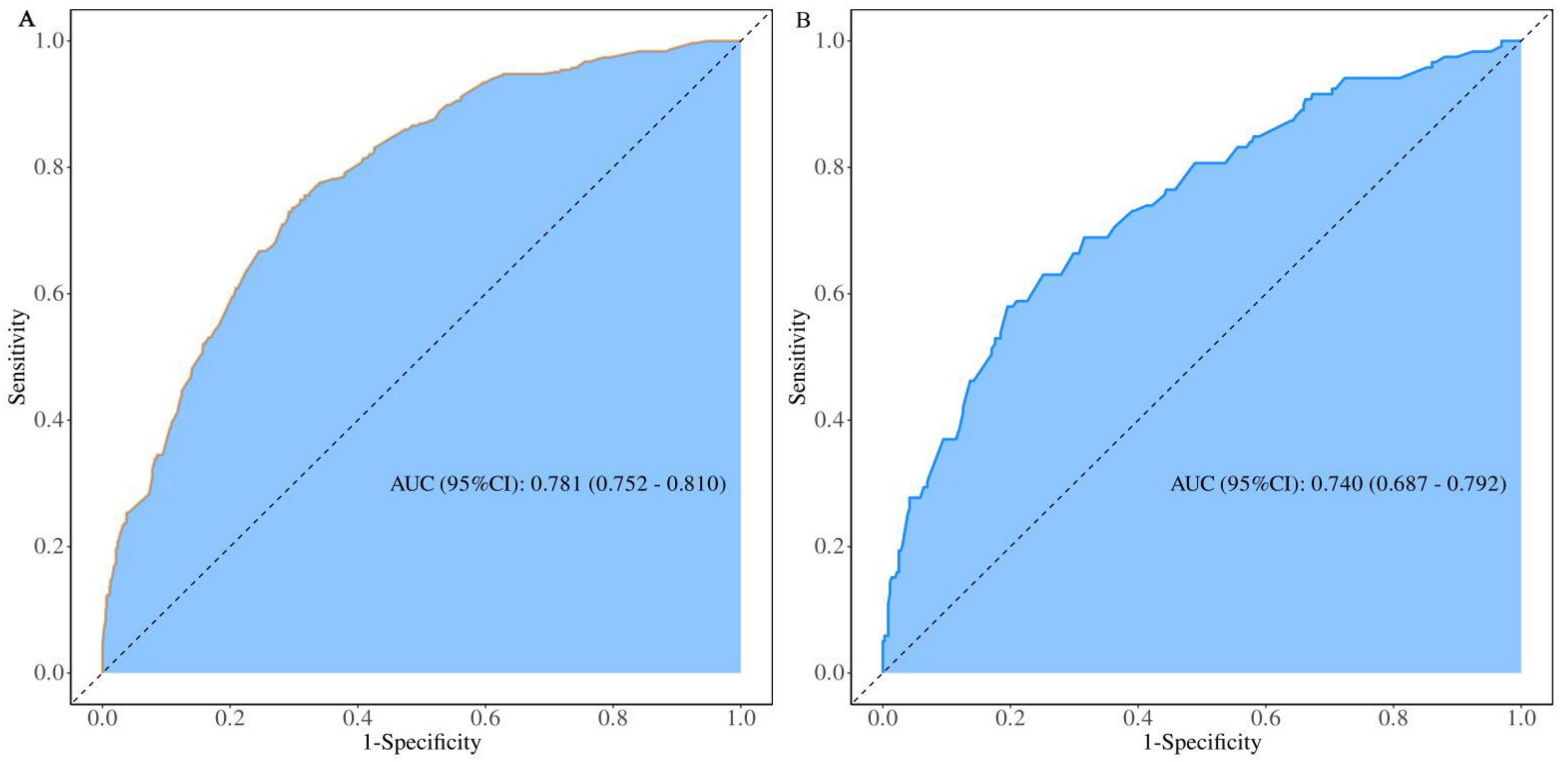
Receiver operating characteristic (ROC) curves of the prediction model for the risk of poor work ability in the training set **(A)** and the validation set **(B)**.

**Figure 4:**
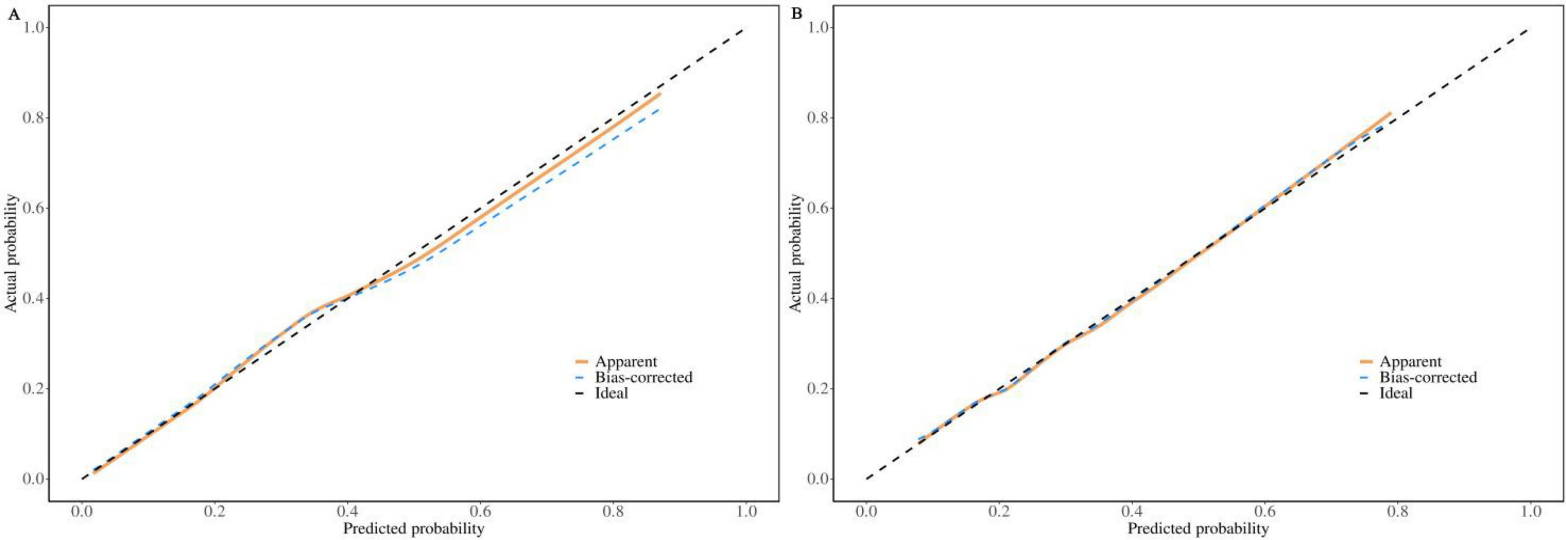
Calibration curves of the nomogram for predicting the risk of poor work ability in the training set **(A)** and the validation set **(B)**.

**Figure 5:**
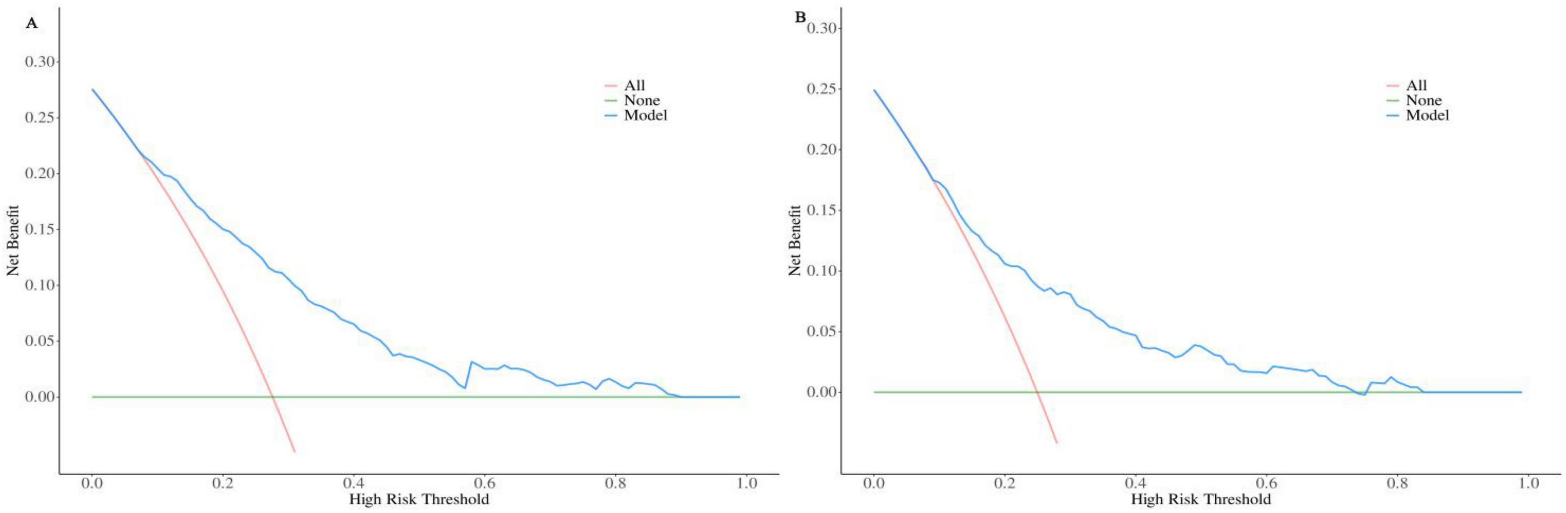
Decision curve analysis (DCA) of the prediction model in the training set **(A)** and validation set **(B)** populations.

In the training set, the nomogram prediction model demonstrated good discriminative ability, with an AUC of 0.781 (95% CI: 0.752–0.810), a sensitivity of 0.756, and a specificity of 0.684. In the validation set, the model’s AUC was 0.740 (95% CI: 0.687–0.792), with a sensitivity of 0.689 and a specificity of 0.656 (Figure 3, Table 5). Calibration curves (Figure 4) showed that the predicted probabilities in both the training and validation sets were in good agreement with the observed probabilities and closely aligned with the ideal diagonal line. The Hosmer-Lemeshow goodness-of-fit test results were not statistically significant (training set: *χ*^2^ = 7.653*, p* = 0.468; validation set: *χ*^2^ = 8.875, *p* = 0.353), indicating good model calibration. Decision curve analysis (DCA) (Figure 5) showed that using this nomogram for risk assessment provided significant net clinical benefit across a wide range of clinical decision thresholds (>3%) for both subsets.

**Table 5:** Performance metrics of the risk prediction model for poor work ability.

Data	AUC (95% CI)	Accuracy (95% CI)	Sensitivity (95% CI)	Specificity (95% CI)	PPV (95% CI)	NPV (95% CI)	Cut off
Train	0.781 (0.752–0.810)	0.704 (0.676–0.730)	0.756 (0.708–0.804)	0.684 (0.652–0.716)	0.476 (0.432–0.521)	0.880 (0.855–0.906)	0.25
Test	0.740 (0.687–0.792)	0.665 (0.620–0.707)	0.689 (0.606–0.772)	0.656 (0.607–0.706)	0.400 (0.333–0.467)	0.864 (0.823–0.905)	0.25

### Interaction effects of factors influencing healthcare workers’ work ability

Logistic regression analysis was performed with work ability as the dependent variable (good = 0, poor = 1), using the group without job burnout and without sleep disorder as the reference. The results (Table 6) showed that, compared to the reference group (7.12% with poor work ability, OR = 1), the OR for poor work ability was 2.890 (95% CI: 2.121–3.939) for those with sleep disorder only, and 3.770 (95% CI: 2.510–5.661) for those with job burnout only. When job burnout and sleep disorder coexisted, the association with poor work ability was significantly stronger (OR = 10.883, 95% CI: 7.048–16.805). Further additive interaction analysis (Table 7) revealed a RERI of 5.164 (95% CI: 2.078–8.251), an AP of 47.45% (95% CI: 29.25–65.65%), and an S of 2.094 (95% CI: 1.377–3.186). This indicates a positive additive interaction between job burnout and sleep disorder on their effect on healthcare workers’ work ability.

**Table 6:** Stratified analysis of job burnout and sleep disorder (*N* = 1,590).

Job burnout	Sleep disorder	Poor work ability (*N*)	Poor work ability (%)	OR (95% CI)
No	No	29	7.13%	1 (Reference)
No	Yes	19	19.59%	2.890 (2.121–3.939)
Yes	No	128	22.98%	3.770 (2.510–5.661)
Yes	Yes	250	47.26%	10.883 (7.048–16.805)

**Table 7:** Additive interaction between job burnout and sleep disorder.

Measure	Estimate	95% CI
RERI	5.164	2.078–8.251
AP (%)	47.453%	29.253–65.653%
S	2.094	1.377–3.186

### Pathway analysis of job burnout, sleep quality, and work ability

#### Model fit results

**Figure 6:**
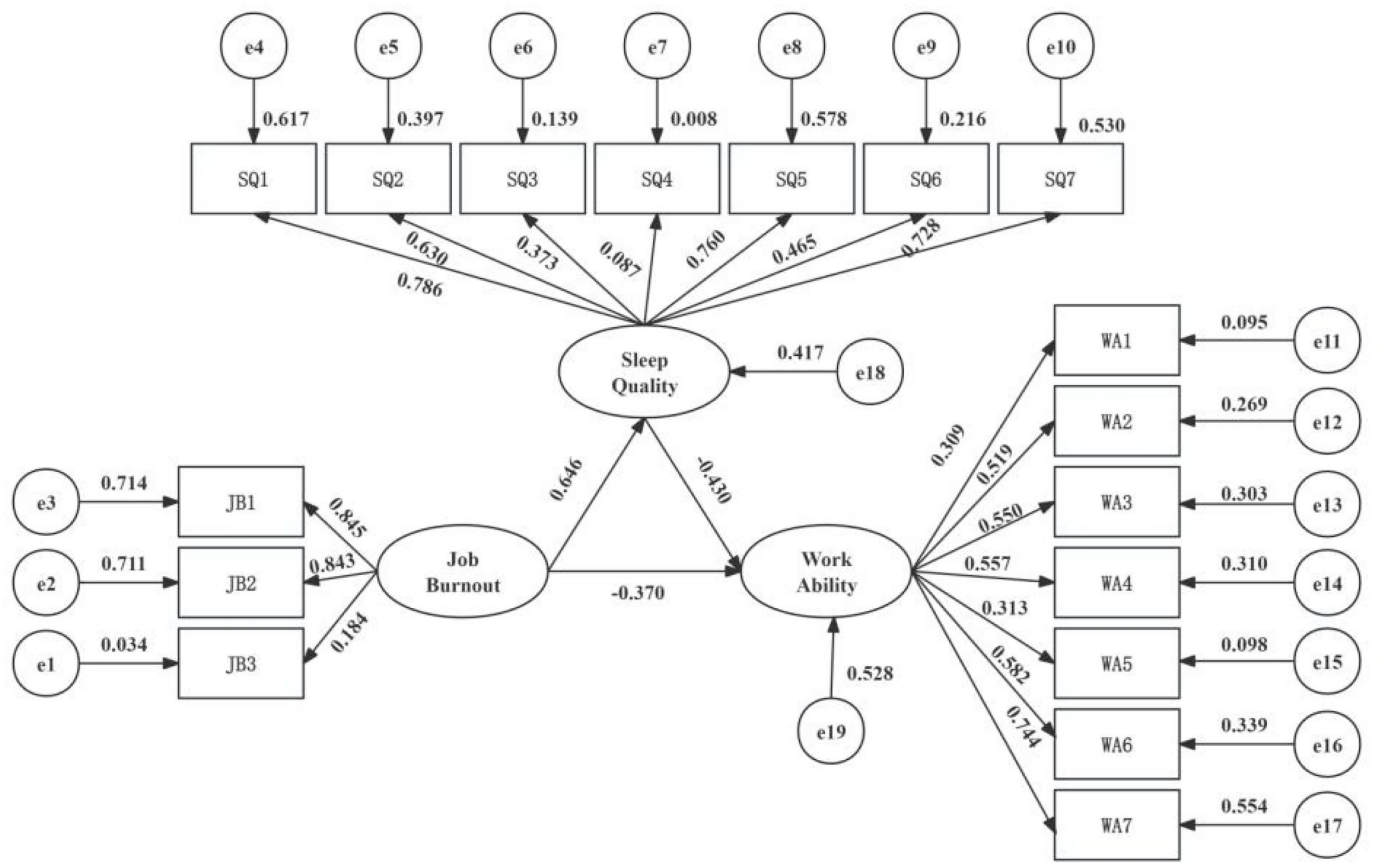
SEM of job burnout, sleep quality, and work ability. SEM of career burnout, sleep quality, and work ability. SEM results for the relationship between burnout, sleep quality, and work ability. All coefficients in the figure are standardized and significant at the 0.05 level. JB1, emotional exhaustion; JB2, depersonalization; JB3, reduced personal accomplishment; SQ1, the subjective sleep quality; SQ2, sleep latency; SQ3, sleep continuity; SQ4, habitual sleep efficiency; SQ5, sleep disorders; SQ6, hypnotic drugs; SQ7, daytime function seven factors; WA1, the lifetime best; WA2, work ability related to professional needs; WA3, the number of current diseases diagnosed by doctors; WA4, estimated work disabilities caused by diseases; WA5, the sick leave in the past year; WA6, the prediction of working ability in 2 years from now; WA7, and mental resources.

A SEM was constructed with job burnout as the exogenous variable and sleep quality and work ability as endogenous variables (Figure 6). The model was fitted using the maximum likelihood method. Given the large sample size (*N* = 1,590), the initial chi-square test of model fit was significant. To control for the influence of the large sample size on goodness-of-fit assessment, the Bollen–Stine bootstrap method was applied for correction (5,000 bootstrap samples) (38, 39). After correction, all model fit indices reached ideal standards (*χ*^2^/df = 1.23, RMSEA = 0.01, CFI = 1.00, etc.; details in Table 8), indicating a good fit between the theoretical model and the data, allowing for pathway analysis.

**Table 8:** Fit indices for the SEM.

Fit index	Initial fit (ML)	Bollen–Stine *P* corrected fit value	Recommended criteria
*χ*2/df	16.08	1.23	<3.0
RMSEA	0.09	0.01	<0.08
GFI	0.79	0.99	>0.90
AGFI	0.84	0.98	>0.90
NFI	0.78	0.99	>0.90
TLI	0.76	1.00	>0.90
IFI	0.79	1.00	>0.90
CFI	0.79	1.00	>0.90

ML = maximum likelihood estimation.

### The influence of job burnout and sleep quality on work ability

SEM analysis indicated that both job burnout and sleep quality were negatively associated with work ability, with standardized path coefficients (*β*) of −0.359 and −0.435, respectively. Furthermore, the association between job burnout and work ability can be partially explained by its association with sleep quality, yielding an indirect effect value of −0.281 (0.645 × −0.435). See Figure 6 and Table 9.

**Table 9:** Path coefficients for the SEM.

Path	Unstandardized estimate	SE	CR	*p*-value	Standardized estimate (*β*)
Job burnout → Sleep quality	1.609	0.262	6.152	<0.01	0.645
Sleep quality → Work ability	−0.426	0.051	−8.400	<0.01	−0.435
Job burnout → Work ability	−0.878	0.170	−5.174	<0.01	−0.359

## Discussion

This study systematically investigated the influencing factors and mechanisms of work ability among healthcare workers in large tertiary hospitals in China by integrating LPA, multivariable regression models, interaction analysis, and SEM. The main findings include: a clear dichotomous latent structure of work ability; job burnout and sleep disorder are key independent predictors of poor work ability and exhibit a significant synergistic interaction; job burnout and work ability may be partially explained by its association with poorer sleep quality; and a multifactorial nomogram model provides a practical tool for clinical risk assessment.

### Heterogeneity in healthcare workers’ work ability: insights from LPA

This study employed LPA, revealing two latent categories of healthcare workers’ work ability with distinct intrinsic attributes: “Good Work Ability” (73%) and “Poor Work Ability” (27%). This not only confirms the heterogeneous structure of work ability within the population, but its high classification accuracy (Entropy = 1.000) further ensures the reliability of the categorization. Of particular importance, an in-depth examination of the profile characteristics reveals qualitative differences that go beyond simple “high versus low scores.” The most prominent features of the “Good Work Ability” category are members’ very positive expectations regarding their own work ability two years into the future, coupled with a lower number of currently diagnosed diseases. This paints a portrait of a “resource-rich and confident” group: they are not only currently in a physical and mental state capable of meeting job demands but also hold an optimistic belief in maintaining this capacity. This positive outlook on the future may itself constitute a key psychological resource, aiding in the enhancement of professional resilience and stress coping. In contrast, the “Poor Work Ability” group exhibits a pessimistic outlook on the future and a higher disease burden, suggesting a lack of confidence in sustainable career development and an exhaustion of health resources. This finding implies that occupational health interventions, while focusing on improving current status, should also strive to cultivate healthcare workers’ sense of control over their career development and an optimistic mindset, thereby interrupting the negative cycle of “loss of confidence → decline in ability.”

### Identification and interpretation of core predictive factors

Multivariate logistic regression analysis indicated that, after controlling for other variables, occupational burnout, sleep disturbances, education level, years of work experience, professional title, and weekly exercise frequency were all statistically associated with the work ability status of healthcare workers. Among these, occupational burnout (OR = 3.770, 95% CI: 2.510–5.661) and sleep disturbances (OR = 2.890, 95% CI: 2.121–3.939) were both positively associated with poor work ability. Previous studies have also suggested a link between occupational burnout and turnover intention among healthcare workers (40). Combined with the findings of this study, occupational burnout is not only closely related to an individual’s immediate work ability status but may also be associated with team stability. This pattern of association suggests that occupational burnout may be a core node requiring systematic attention in occupational health management. From a theoretical mechanism perspective, the relationship between sleep disturbances and work ability can be partly explained from the perspective of cognitive resources. Research evidence indicates that sleep problems are associated with impairments in fundamental cognitive abilities, including executive function and working memory (41). For healthcare workers engaged in high-demand clinical tasks, such depletion of cognitive resources is likely reflected in comprehensive assessments of their overall work ability (such as that measured by the WAI), since such evaluations inherently incorporate considerations of mental efficiency and cognitive adaptability.

The multivariable regression analysis in this study indicated that a higher education level had a protective association with healthcare workers’ work ability. Compared to those with an associate degree or below, individuals with a master’s degree (OR = 0.371, 95% CI: 0.195–0.708) or a doctoral degree (OR = 0.216, 95% CI: 0.068–0.684) had a significantly reduced risk of poor work ability. This finding suggests that educational attainment, as a comprehensive reflection of an individual’s knowledge integration and professional skill level, may be an important resource for maintaining good work ability. Compared to healthcare workers with ≤5 working years, those with 6–10 working years had a lower risk of poor work ability (OR = 0.647, 95% CI: 0.425–0.986). This may be because their professional skills have become proficient, while burnout resulting from long-term occupational stress has not yet peaked, placing them in a stage of relative balance between professional efficacy and stress. However, working 21–30 years (OR = 2.417, 95% CI: 1.345–4.344) and ≥31 years (OR = 2.548, 95% CI: 1.116–5.818) were associated with a significantly increased risk of poor work ability. This likely reflects the combined effect of multiple challenges, including the cumulative impact of long-term occupational stress, physiological changes associated with aging, and potential career plateaus (42, 43). The analysis of professional title association showed that the strength of association between intermediate and senior titles and poor work ability status was significantly higher than that for junior titles (intermediate: OR = 1.789, 95% CI: 1.132–2.829; senior: OR = 2.134, 95% CI: 1.091–4.175). This positive association may stem from the fact that promotion often entails heavier combined clinical, teaching, research, and administrative workloads. The increased responsibility pressure coupled with structural constraints on work autonomy may be the underlying mechanism (44, 45).

This study found a significant association between physical exercise frequency and work ability status. Compared to those who did not exercise, healthcare workers who exercised 1–2 times per week (OR = 0.570, 95% CI: 0.407–0.798) and more than 5 times per week (OR = 0.287, 95% CI: 0.128–0.642) were less likely to have poor work ability. A potential explanation for this association may be that regular exercisers generally possess better physical fitness reserves, and their stress regulation systems (e.g., cortisol levels) and neuroendocrine functions (e.g., endorphin release) may be in a more favorable state. These factors are often associated with better mood and sleep experiences (46, 47). To translate the research findings into a practical tool, a clinical prediction nomogram was constructed based on the aforementioned multivariable model. This model integrates six key predictive indicators, enabling a quantitative assessment of the risk of work ability decline for individual healthcare workers. Internal validation showed good model discrimination (training set AUC = 0.781, 95% CI: 0.752–0.810; validation set AUC = 0.740, 95% CI: 0.687–0.792). Calibration curves and decision curve analysis (DCA) confirmed its predictive accuracy and clinical utility, respectively. The value of this tool lies in its ability to help hospital administrators conveniently identify high-risk individuals, thereby promoting a shift in occupational health management from a “universal intervention” model to a “risk-based precision prevention” model, such as prioritizing comprehensive interventions for burnout management and sleep promotion for those with high scores.

### Synergistic effect and influential pathways of job burnout and sleep disorder

One of the most salient findings of this study is the revelation of a positive additive interaction between job burnout and sleep disorder on work ability. When both conditions coexist, their combined effect (OR = 10.883) is statistically significantly greater than the sum of their independent effects (RERI = 5.164, AP = 47.45%). This result provides strong support for the “loss spiral” hypothesis derived from the Conservation of Resources theory (7). When job burnout (depletion of psychological resources) and sleep disorder (impairment of physiological restorative resources) coexist, individuals are trapped in a dual dilemma of “accelerated resource depletion” and “severely hindered recovery.” The overall scarcity of resources makes it difficult for them to adopt effective strategies to address either problem, leading to a situation where the two conditions become mutually reinforcing and locked in a vicious cycle, inflicting a synergistic ‘1 + 1 > 2’ destructive impact on work ability. This statistically confirms the real-world existence of the “loss spiral” in the realm of workplace health. SEM indicated a direct negative association between job burnout and work ability (*β* = −0.359, *p* < 0.01), as well as a significant indirect association pathway mediated through sleep quality (indirect effect *β* = −0.281, *p* < 0.01). This suggests that a substantial portion of the negative impact of job burnout on work ability is realized through the chained resource loss of triggering or exacerbating sleep disorder. This pathway of “psychological resource depletion → impaired physiological restoration → decline in work ability” perfectly exemplifies the core tenet of the Conservation of Resources theory that “the loss of key resources begets the loss of secondary resources” (7).

Integrating the findings from both the interaction effect and the mediation effect, this study, from the perspective of cross-sectional data, delineates a picture consistent with the dynamic “loss spiral” described by the Conservation of Resources theory: job burnout and sleep disorder can either act in synergy to deliver a combined blow to work ability, or they can link sequentially to form a channel for the gradual attrition of resources. Although the causal temporal sequence requires confirmation through longitudinal studies, this integrated model provides a coherent theoretical explanation for understanding the complex, cumulative process of occupational health risks among healthcare workers.

Based on the aforementioned findings, the nomogram model constructed in this study provides a practical tool for identifying high-risk individuals. Management practices should shift toward an integrated intervention strategy that equally emphasizes “resource replenishment” and “loss interruption”: on one hand, directly supplementing psychological resources and alleviating burnout through means such as organizational support and cognitive-behavioral therapy; on the other hand, it is essential to elevate sleep health promotion as a core strategy, protecting and repairing physiological restorative resources by improving shift schedules, providing sleep hygiene education, etc., thereby interrupting the “loss spiral.” Future research should validate this model in multi-center samples and, through longitudinal or interventional designs, directly test the effectiveness of interventions based on the Conservation of Resources theory in blocking the “loss spiral” and enhancing work ability.

### Study limitations

Despite obtaining a series of meaningful findings through the integration of multiple statistical methods, this study has several limitations that warrant careful consideration when interpreting the conclusions and planning future research directions.

First, regarding study design. This study employed a cross-sectional design, with all data collected at a single time point, which limits inferences regarding the causal direction and dynamic temporal sequence among variables (such as between job burnout, sleep disorder, and work ability). Future longitudinal studies or intervention experiments are crucial for verifying causal relationships among them.

Second, concerning the sample and data collection. Firstly, the data were sourced from a single large tertiary A-level hospital in Shaanxi Province, China, using convenience sampling. Healthcare workers in this hospital have specific characteristics regarding workload, resource allocation, management models, and regional culture. Therefore, caution is warranted when generalizing the study’s conclusions to other regions within China or to healthcare institutions at different levels or with different management models. Secondly, this study collected data using self-report questionnaires, which may be susceptible to social desirability bias, recall bias, and common method bias, although anonymity was employed to partially mitigate social desirability effects.

Third, on the methodological level. The analytical strategy of this study encompassed two paths: Firstly, when exploring the associations and interaction effects between predictors and work ability categories, a two-step “classify-analyze” approach was adopted—namely, first classifying work ability through LPA and then using this classification result as the dependent or grouping variable for subsequent logistic regression and interaction analysis. Methodological research indicates that such two-step approaches may introduce parameter estimation bias when latent class classification is unclear (i.e., has low entropy). With respect to the LPA classification itself, the classification clarity in this study was extremely high (entropy value = 1.000), which substantially reduces the risk of bias in logistic regression and interaction analyses due to ambiguous classification. Nevertheless, it is important to note that future researchers employing a similar “classify-analyze” strategy should interpret inferences based on classification results cautiously if their data exhibit insufficient classification clarity and should consider using more integrated modeling approaches.

Based on the above limitations, future research can be deepened in several directions: enhancing sample representativeness through multi-center, multi-level sampling designs; employing longitudinal or experimental designs to verify causal relationships between variables; supplementing self-report data with objective measurement indicators (e.g., physiological data, institutional records); and, when conditions permit, applying more complex integrated statistical models for direct testing of theoretical mechanisms.

## Conclusions and recommendations

This study systematically revealed the mechanisms influencing the work ability of healthcare workers in large tertiary hospitals in China. Job burnout and sleep disorder are two core, intervenable targets, and they exhibit a hazardous synergistic effect. Longer working years and higher professional titles were positively associated with poor work ability, whereas higher educational attainment and regular physical exercise were negatively associated with poor work ability.

Based on these findings, we propose the following recommendations: At the organizational level, hospital administrators should integrate job burnout and sleep health into a systematic occupational health management system, conduct regular screenings, and provide integrated interventions (such as cognitive behavioral therapy, mindfulness-based stress reduction, and sleep hygiene education) for identified high-risk groups. At the practical level, encourage and create conditions for physical exercise, and utilize the work ability risk assessment tool for employee health record management to achieve precise prevention. Future research should conduct longitudinal or interventional studies to verify causal relationships and perform external validation in multi-center, multi-level healthcare institutions to establish more generalizable risk prevention and control strategies.

## Data Availability

The raw data supporting the conclusions of this article will be made available by the authors, without undue reservation.
